# Umbilical Venous Catheters and Peripherally Inserted Central Catheters: Are They Equally Safe in VLBW Infants? A Non-Randomized Single Center Study

**DOI:** 10.3390/medicina55080442

**Published:** 2019-08-06

**Authors:** Aikaterini Konstantinidi, Rozeta Sokou, Polytimi Panagiotounakou, Maria Lampridou, Stavroula Parastatidou, Katerina Tsantila, Eleni Gounari, Antonios K. Gounaris

**Affiliations:** 1 NICU, General Hospital “Agios Panteleimon”, 18454 Piraeus, Greece; 2 Royal Alexandra Children’s Hospital Brighton, Eastern Road, Brighton, East Sussex BN2 5BE, UK; 3 NICU, University General Hospital, 41222 Larissa, Greece

**Keywords:** very low birth weight neonates, central venous catheters, umbilical venous catheters, peripherally inserted central catheters, central lines associated bloodstream infection

## Abstract

*Background and Objective:* Peripherally inserted central catheters (PICC) and umbilical venous catheters (UVC) are frequently used for vascular access in neonatal intensive care units (NICUs). While there is a significant need for these devices for critically ill neonates, there are many complications associated with their use. We aimed at investigating the incidence of UVC and PICC complications in very low birth weight (VLBW) infants. *Materials and Methods:* This is an observational study performed with neonates of the tertiary General Hospital of Piraeus, Greece, during an 18 month-period. Seventy-one neonates were recruited and divided into two groups: 34 neonates with PICC and 37 neonates with UVC. We recorded: Catheter dwell time, the causes of catheter removal, other complications, infections, and catheter tip colonization rates. *Results*: No significant statistical differences were noticed between the 2 study groups with regards to demographic characteristics, causes for catheter removal, catheter indwelling time or the incidence of nosocomial infection. Eleven UVC tips and no PICC tips were proved colonized (*p* = 0.001) following catheter removal. *Conclusions:* The incidence of complications associated with the use of UVCs and PICCs in VLBW infants did not significantly differ in our study. Their use seems to be equally safe. Further studies, with larger samples, are necessary to confirm our results.

## 1. Introduction

Intravenous lines are broadly divided into two categories: Short-term peripheral lines and central venous catheters (CVCs). CVCs are essential in neonatal intensive care units (NICUs) for diagnostic and therapeutic purposes in neonates. They are commonly used to provide fluids, medications and total parenteral nutrition (TPN) to critically ill infants, especially to very low birth weight (VLBW) and extremely low birth weight (ELBW) neonates, in line with the practice of minimal handling, allowing longer dwell time and less frequent need for reinsertion, thus reducing stress [1,2]. Central catheters are recommended in view of convenience, cost-benefit, and fewer complications than peripheral short-term venous catheters [2,3], and skin tunneled cuffed catheters (Hickman, Broviac) [4,5,6,7]. The commonly used CVCs in the NICU are eripherally inserted central catheters (PICCs) or “long lines” and umbilical venous catheters (UVCs).

A PICC is inserted in one of the major peripheral veins. Insertion can take place on the first day of life (following failure of UVC insertion) or at any time during the entire NICU stay. Common sites of PICC insertion are the veins of the upper limbs (basilar cephalic, axillary, veins of the forearm), of the lower limbs (small saphenous vein at the level of popliteal vein, or great saphenous vein at the level of internal malleolus) and rarely, others, such as the external jugular and posterior auricular vein. Panagiotounakou et al. [8], randomized 61 premature infants to receive PICC inserted in an axillary vein or other areas further away (or distally). PICCs inserted in the axillary vein were 12 times less likely to have complications and 7 times more likely to avoid early removal due to complications.

For upper extremities, the optimal line tip position is in the superior vena cava (for preterm neonates 0.5–1 cm outside the cardiac chambers and for term neonates 1–2 cm outside the cardiac chambers) and for lower extremities, inside the inferior vena cava. However, due to technical challenges, a significant number of catheters may end up with their tip in suboptimal non-central positions; for example, for the catheters with their tip in subclavian veins, which were termed midclavicular, no complications were noted [9,10]. The optimal position of the UVC is at the junction of the inferior vena cava and right atrium, above the diaphragm (Τ8–Τ10) [11].

Both of these catheters are associated with multiple and often severe complications, including infection, local edema, thrombosis [12], blockage, displacement, liver abscess, pericardial effusion/tamponade, portal venous thrombosis, pleural effusion, embolization, and non-elective removal [9,13,14,15]. Evidence in the literature shows that the use of UVC followed by PICC in VLBW neonates, is superior compared to peripheral venous access only [16]. One RCT conducted in India, examined UVCs and PICCs in terms of success rate, complications, cost, and time of insertion in NICU and concluded that UVC is a cheaper alternative to PICC, with similar success rate, short-term complications and time needed for insertion [6].

Unit protocols or clinician preferences may guide the choice of one of the two CVCs. In most NICUs, UVCs are not used for more than 5–7 days, due to increased incidence of late onset sepsis (LOS) related to UVC longevity [17]. The use of PICCs in VLBW infants has been investigated in various prospective, retrospective studies and meta-analyses [2,18] and is considered safe, while research concerning UVCs use is scarce [17].

The main objective of our study was to compare the incidence of complications between the two types of central venous catheters (UVCs vs. PICCs) and evaluate the safety of their use for as long as their support is needed. The secondary objective was to investigate and analyze: (1) The nature of complications, (2) the reasons for line removal, (3) catheter dwell time, (4) infection and (5) catheter tip colonization.

## 2. Methods

### 2.1. Study Design-Setting and Ethical Approval

This observational study was conducted during an 18 month period at a tertiary-level 26 bed NICU in Greece with average NICU admissions of 400 infants per year. The study was conducted in accordance with the Declaration of Helsinki, and the protocol was approved by the Ethics Committee of the General Hospital of Nikaia-Piraeus ‘Aghios Panteleimon’ (Project identification code 23.05.2007, 30/11). Parental informed consent was obtained for all neonates included in the study.

### 2.2. Participants

During this time period, a CVC (either UVC or PICC) was inserted in 71 VLBW neonates hospitalized in our NICU. The study group population was divided into two groups depending on the type of venous catheter that was finally inserted: Group A consisted of infants with PICCs (because of UVC insertion failure during the first 3 days of life) and group B consisted of infants that had only UVC and PICC never was inserted.

Inclusion criteria were: (1) Birth weight below 1500 g and gestational age < 32 weeks. Gestational age was defined by strict criteria, prioritizing menstrual dating confirmed by early ultrasound. (2) Insertion of CVC (UVC or PICC) in our NICU.

Exclusion criteria included: (1) Catheter removal within 24 h following insertion because of inappropriate line tip position, as the complication rate was expected to be low due to the short indwelling time; (2) CVC insertion in another center, because of possible differences or incomplete data regarding the insertion procedure that might affect the complication rate; (3) congenital abnormalitie; and (4) necrotizing enterocolitis (NEC) Bell stage II or III, during the first five days of life.

No randomization was performed, as the type of catheter (UVC or PICC) placed in the neonates was based on the protocol of our unit. In VLBWs infants scheduled for a long NICU hospitalization, the preferred option was catheter insertion in the umbilical vein on the first or second day of life. In case the first UVC insertion attempt in the inferior vena cava failed or in case of early UVC catheter removal due to various reasons, a PICC insertion was performed, usually after the third day of life.

Skin antiseptic preparation included cleansing the site of the planned procedure three times with a cotton swab remoistened with the antiseptic solution, povidone-iodine 10%. To avoid prolonged exposure to iodine, skin sites disinfected with povidone-iodine were wiped with sterile normal saline solution after 60 s until all antiseptic stains were removed [19]. The approximate size of the disinfected area was 7 cm diameter for insertion of UVC and PICC. The distal edge of the catheter was disinfected with a 0.5% chlorhexidine/alcohol 70% solution at least three times daily, according to the instructions of the Infectious Diseases Committee of our Hospital.

UVC access (with single-lumen umbilical catheters, PUR, 2.5, 3.5, 5Fr, Vygon, Ecouen, France) of the inferior vena cava was performed by a group of trained neonatologists within the incubator, under sterile conditions.

The tip of the UVC was positioned in the inferior vena cava just before the junction with the right atrium, above the level of the diaphragm. The catheter was secured in place with a suture through the umbilical cord stump. An X-ray was performed and UVC was secured using goal posts. Umbilical stump (not skin) was cleaned with aqueous chlorhexidine 0.015% solution. The catheter was further secured using ‘H-Tapes’ (leucoplast brown) affixed to the abdominal wall. ‘H-Tapes’ must secure the catheter against movement or accidental dislodgement, which can result in loss of vascular access and significant blood loss. Nurses checked for loose ‘H-Tapes,’ due to the humidified environment, which is necessary for neonates with birth weight <1500 g.

The PICC (Nutriline 2Fr-24G, Vygon, Aachen, Germany) insertion was performed during the morning shift by a trained group of neonatologists and nurses. The same group was also responsible for infant monitoring and catheter removal. Catheter insertion was performed within the incubator, under sterile conditions. PICC was inserted in one of the major peripheral veins (i.e., axillary vein, basilic vein, cephalic vein, greater saphenous vein, or temporal vein) and advanced to the junction between the superior or the inferior vena cava and the right atrium.

**Figure 1:**
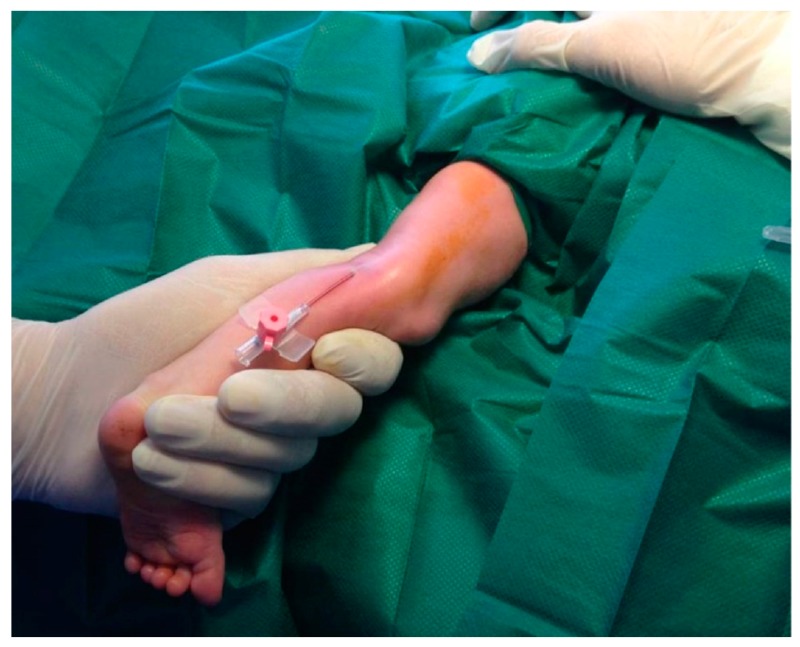
Venipuncture performed with a 20G regular venous catheter.

Venipuncture was not performed with the 20G butterfly needle included in the kit of the radiopaque catheter, since it caused severe local tissue damage and hemorrhaging. Instead, we used a 20G regular venous catheter with a modified technique (Figure 1): After successful venipuncture, the needle of the regular venous catheter was carefully removed to maintain patency of the vein.

**Figure 2:**
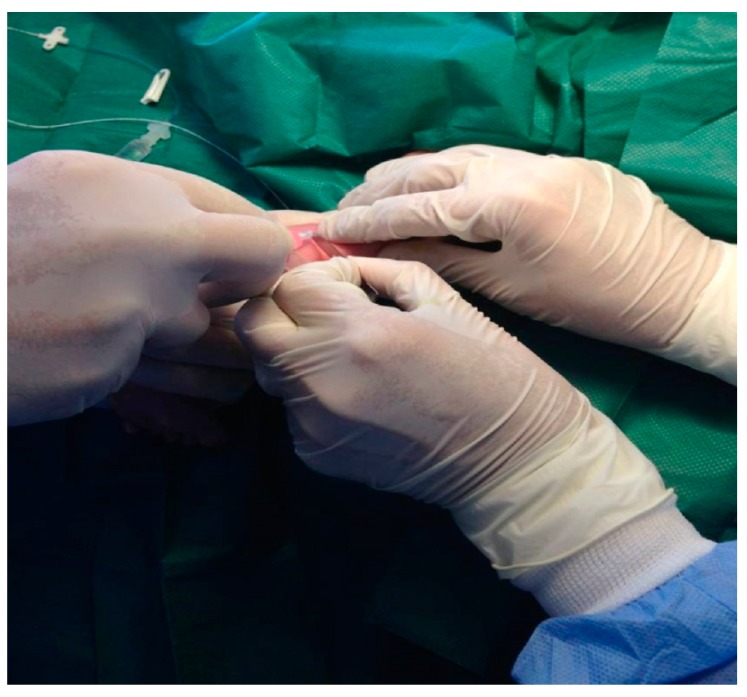
The tube of the regular venous catheter is cut at the site of the beck.

The tube of the regular venous catheter was cut at the site of the beck (Figure 2) and was used as a guide for the insertion of the PICC catheter which was carefully advanced into the vessel, at the desired length, estimated on the skin beforehand. Sometimes we had to move and lift the corresponding limb of the baby and so facilitate advancing the catheter line.

**Figure 3:**
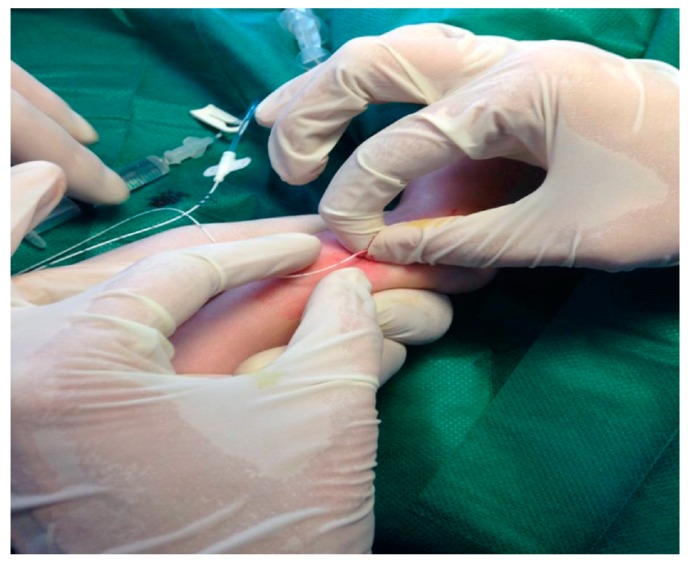
PICC catheter advancement through the tube that serves as a guide.

Once the central catheter was in place, the tube of the regular venous catheter was slowly removed from the vein and pulled along to the length of the PICC catheter towards its distant edge (Figure 3). Finally we covered PICC catheter at the insertion site using a sterile transparent film dressing (Tegaderm, 3M Health Care, St. Paul, MN, USA). This dressing was changed every week or whenever its change was deemed necessary.

Correct placement of CVC tip was confirmed by an X-ray, according to the literature [11,20,21], before administering TPN or drugs, in order to avoid complications of inappropriate positioning of the catheter. Heparin was added to the TPN using a sterile technique at a concentration of 0.5 IU/mL [22]. Normal saline 0.9%, was used before and after the administration of antibiotics or blood products, up to an amount of 3 mL with a 10 mL syringe, to keep the patency of CVC. Nursing procedures for maintaining line sterility over time were preformed according to “Aseptic Non Touch Technique” which includes: Hand hygiene, sterile field selection, glove use, hub disinfectant, and preparation of equipment using a non-touch technique. Prior to use, a needleless connector was cleaned for 15 s using an alcohol swab and friction in a twisting motion and dried. Tubing and extension sets were changed every 24 h. Following transfusions with blood products, the extension piece and needleless connector were changed.

Whenever a neonate presented with clinical signs or symptoms of sepsis, blood culture was performed prior to antibiotic therapy initiation. Blood specimens were collected through peripheral venipuncture, on separate occasions: From at least two separate blood draws on the same or consecutive calendar days, or two separate site preparations (decontamination steps) performed during specimen collection. No blood specimens were drawn through central catheters.

Both venous catheters remained in site until full enteral feeding of the infant or until the development of any complications. Parenteral nutrition, antibiotics, inotropic drugs and plasma were administered through UVCs and PICCs, whereas condensed red blood cells were administered only through UVCs. All infants with UVCs were monitored weekly with liver ultrasound for portal vein thrombosis. In our NICU, all neonates with CVCs also underwent echocardiogram. We electively removed UVC or PICC when the infants were fully fed, or antibiotic therapy was not necessary anymore. At the time of catheter removal, the catheter tip was collected under aseptic conditions. The distal end of the catheter was cut with sterile scissors, placed into sterile container and taken to the laboratory as soon as possible. Each catheter tip was cultured by semi quantitative (roll plate) method on blood agar media. After a 24 h incubation, the finding of more than 15 colony forming units (CFUs) of bacteria in culture was considered as catheter tip colonization.

### 2.3. Recorded Data

For all study newborns we recorded: (1) Demographic data; (2) catheter dwell time; (3) the causes of catheter removal; (4) other complications, such as portal vein thrombosis-obstruction for UVCs, and edema-erythema-obstruction-thrombosis for PICC; (5) infection; and (6) catheter tip colonization rate.

In our study, nosocomial infection was defined using one of the following definitions:

1. Central lines associated bloodstream infection (CLABSI) according to CDC definition: Presence of bacteria in a single blood culture (for organism not commonly present on the skin), or in two or more blood cultures (for organisms commonly present on the skin), obtained from a symptomatic infant either within 48 h after a central catheter insertion or within a 48-h period following catheter removal, and not related to an infection at another site [19,23,24,25].
2. Probable but unproven sepsis, based either on clinical signs (aggravated clinical status presenting with apnea, hyperthermia or hypothermia, tachycardia or bradycardia, hypotension, hyperglycaemia), and/or on laboratory findings (elevated C-reactive protein along with two of the following: Immature/mature white blood cell ratio > 0.2, low (<100,000) platelet count, neutrophils white blood cell count of <1500 without positive blood culture, and being defined as a systemic condition resulting from an adverse reaction to the presence of an infectious agent that was neither present nor incubating at the time of admission to the hospital [26].

### 2.4. Statistical Analysis

SPSS 17.0 version for Windows (SPSS Inc., Chicago, USA) was used for statistical analysis. The incidence of infection was measured as infection episodes per 1000 catheter-days. Chi-squared tests were performed for categorical variables, *t*-tests for independent samples and Mann-Whitney tests for nonparametric variables. We performed binary logistic regression to calculate odds ratios. A *p*-value of <0.05 was considered significant.

## 3. Results

During the 18-month study period, CVCs were inserted (34 PICCs and 37 UVCs) in 71 neonates hospitalized in our NICU. Clinical characteristics of the infants are presented in Table 1.

Causes of catheter removal in the two study groups are presented in Table 2. No significant statistical differences were noticed between the two study groups with regards to demographic characteristics or causes for catheter removal.

One of the newborns with CLABSI, in Group B (UVC group of neonates,), was diagnosed with portal vein thrombosis, confirmed by ultrasound on the 12th day of hospitalization. The UVC was removed and the neonate was initially treated with alteplase and afterwards with low-molecular-heparin for 6 months. Regarding catheter tip colonization, no catheter tip (PICC) was found colonized in group A (PICC group of neonates), compared with 11 (29.72%) catheter tips (UVCs) in group B (four of them were removed because of nosocomial infection, the three out of four had probable but unproven sepsis). The difference between the two groups was statistically significant (*p* = 0.001).

We found that the incidence of nosocomial infection increases by 1.38 times ((95% C.I. 0.983–1.948), *p* = 0.063) per day of catheterization for UVC use, and 1.01 times ((95% C.I. 0.851–1.198), *p* = 0.911) for PICC use.

## 4. Discussion

This observational study did not reveal any statistically significant differences between complication rates of UVCs and PICCs in VLBW newborns younger than 32 weeks of gestation. To the best of our knowledge, this is one of few reports that include an analysis of all kind of UVC and PICC complications in a NICU during an 18-month period.

Shalabi et al. [27] published a retrospective matched cohort study using data from 29 of the 30 tertiary-level NICUs in Canada, in which no significant difference was found in the incidence of CLABSI between very preterm neonates who received a PICC, UVC, or UVC followed by PICC as the primary mode of venous access after birth. In our study, we investigated not only CLABSI, but all possible complications associated with CVCs use in VLBW infants. Another difference between the two studies is that our data were selected from one-single-center with the same practices regarding the insertion procedure and surveillance, thus reducing possible differences that might have an impact on the complication rate.

PICCs and UVCs used in VLBW infants, and especially in ELBW infants, involve complications [28,29]; however, they cause less disturbance and stress than peripheral venous catheters [1,30] or CVCs such as Broviac [5].

According to the literature, the most common complication leading to catheter removal is CLABSI or blood stream infection [31,32,33]. The incidence of CLABSI varies from 2.1 to 17 per 1000 catheter days or 6% to 36.8% depending on the definition of CLABSI [31]. In our study, the incidence of nosocomial infection was 6.06 (7%), and when CLABSI was defined according to the CDC/National Healthcare Safety Network (NHSN) definition, our CLABSI incidence was lower (2.42 (2.8%) per 1000 CVC days) [33]. Moreover, although the incidence of nosocomial infection increases per day of catheterization for both catheters use, this finding was not statistically significant. Nosocomial infection is associated with high mortality and serious morbidity in preterm neonates.

Literature data show that the use of impregnating or coating central venous catheters with anti-infective agents, including antiseptics and antibiotics, reduce catheter related bloodstream infection in adults and children receiving intensive care, but there is a paucity of similar evidence for babies receiving neonatal intensive care [34,35].

Shalabi M. et al. [27] suggested that the use of UVCs in preterm infants is associated with an increased rate of LOS after a median period of 5 days, and an alternative access point is usually needed after UVC removal.

In our study, despite a longer UVC indwelling time (3–25 days), the incidence of CLABSI was 2.7% (2.59 per 1000 UVC days), and there was no statistical difference compared to PICC use which was 2.9% (2.28 per 1000 PICC days).

Complications associated with mal-position of PICCs and UVCs, although rare, may evolve to life threatening situations. Misplacement of a PICC can lead to the development of pleural effusion, perforation of the vessel, etc. [36,37,38]. On the other hand, misplacement of a UVC out of the inferior vena cava may cause liver injury (which often leads to elevated liver enzymes), portal hypertension, hepatic necrosis, and/or effusions [39,40,41]. Extremely deep placement of the catheters can cause heart and lung injuries, ranging from transient edema to pulmonary hemorrhage, and pleural or pericardial effusion [14,42,43]. Fortunately, no such complications were recorded in our study.

The incidence of complications, such as edema, erythema, and obstruction for PICCs and thrombosis for UVCs, was similar to those described in the literature [32].

It is important to note the difference in tip colonization rates between the two catheter types (*p* = 0.001) though it did not cause clinical deterioration to the neonates. No convincing interpretation was identified for this difference. The colonization of the umbilical stump by microorganisms [31] after the first days of life and the long UVC indwell time could probably give an explanation. The more frequently isolated species were several types of staphylococci (80%). Cronin et al. [28], evaluated intravascular tip colonization in critically ill neonates, in relationship to the type of device used and found 14% of UVCs colonized. This rate of tip colonization is almost half, compared to ours which was 27.9%, probably due to shorter UVC dwelling time. Except from the above mentioned difference, overall complication rate was the same for the two groups.

The most important limitations of this study include the small, one-center sample size and the fact that power analysis was not performed. On the other hand, we believe that using data from a single center minimizes the effects of different practices and incomplete data regarding the insertion procedure and monitoring, that might affect the complication rate.

## 5. Conclusions

Our results show that both CVC types, UVC and PICC are similar in complication rates when using in VLBW infants at this setting. UVCs and PICCs can be used in VLBW neonates for as long as their support in administration of parenteral nutrition and drugs is required. The prevention of CVC’s complications should be an important goal in the daily care of neonates in NICU. Further research would be helpful to confirm our results, thus decreasing the complications associated with CVCs. Efforts to remove the CVCs at the earliest possible time should be a goal for neonatologists and nurses in NICUs.

## Figures and Tables

**Table 1:** Characteristics of study neonates.

	PICC *n* = 34 (47.89%)	UVC *n* = 37 (52.11%)	*t*-Test	Mann-Whitney Whitney U
Birth weight (grams)	1034 ± 214	1041 ± 179	*p* = 0.89	
Gestational age (weeks)	28.7 ± 2.3	28.5 ± 1.99	*p* = 0.79	
CVC indwelling time (days)	11.91 ± 6.93 (median = 11.5) (range: 3–31)	10.43 ± 5.38 (median = 11) (range: 3–25)		*p* = 0.152

Note: PICC = peripheral inserted central catheter, UVC = umbilical venous catheter, CVC = central venous catheter. Values are mean ± standard deviation.

**Table 2:** Reasons for central venous catheter (CVC) removal.

	Total *n* = 71 (100%)	PICC *n* = 34 (47.89%)	UVC *n* = 37 (52.11%)	Chi-Squared Test
End of treatment	62 (87.3%)	31 (91.2%)	31 (83.8%)	*p* = 0.061
CLABSI	2 (2.8%)	1 (2.9%)	1 (2.7%)	*p* = 0.952
	2.42 per 1000 CVC days	2.28 per 1000 PICC days	2.59 per 1000 UVC days	
Nosocomial infection	5 (7%)	1 (2.9%)	4 (10.8%)	*p* = 0.195
	6.06 per 1000 CVC days	2.28 per 1000 PICC days	10.3 per 1000 UVC days	
Obstruction	1 (1.4%)	1 (2.9%)	-	N/A
Local edema + Skin irritation	2 (2.8%)	2 (5.88%)	-	N/A
Skin irritation	1 (1.4%)	1 (2.9%)		N/A
Accidental removal	2 (2.8%)	-	2 (5.4%)	N/A
Total of complications	11 (15.5%)	5 (14.7%)	6 (16.2%)	*p* = 0.861

Note: PICC = peripheral inserted central catheter, UVC = umbilical venous catheter, CVC = central venous catheter. CLABSI = central lines associated bloodstream infection. NA: Not applicable. Total CVC days: 825, total PICC days: 439, total UVC days: 386.
